# Neurocognitive and emotional long-term effects of COVID-19 infections in children and adolescents: results from a clinical survey in Bavaria, Germany

**DOI:** 10.1186/s12879-025-10813-w

**Published:** 2025-03-26

**Authors:** Julia Hauke-Gleißner, Irina Jarvers, Silke Jordan, Stephan Gerling, Michael Kabesch, Romuald Brunner, Stephanie Kandsperger

**Affiliations:** 1 Department of Child and Adolescent Psychiatry and Psychotherapy, University of Regensburg, Regensburg, Germany; 2 University Children’s Hospital Regensburg (KUNO) at the Hospital St. Hedwig of the Order of St. John, University of Regensburg, Regensburg, Germany; 3 Research and Development Campus Regensburg (WECARE) at the Hospital St. Hedwig of the Order of St. John, Regensburg, Germany

**Keywords:** post-COVID syndrome, Children, Adolescents, Neurocognition, Emotional problems, Behavioral problems, Long-term effects, Risk factors, Psychiatric disorders

## Abstract

**Background:**

While children and adolescents typically experience mild symptoms during the acute phase of the COVID-19 infection, some may develop severe post-infectious symptoms. In our study *Post-COVID Kids Bavaria* we integrated somatic and psychiatric aspects of the post-COVID syndrome to provide a holistic description of symptoms, provide early treatment, and detect possible risk factors associated with post-infectious neurocognitive and emotional impairments.

**Methods:**

We conducted an observational study involving 85 pediatric patients aged 12–17 years (M = 12.48, 61.2% female) who had confirmed COVID-19 infections and were experiencing persistent symptoms for at least 4 weeks. Our neuropsychological assessment comprised infection-specific patient interviews, psychopathological examinations, emotional well-being and behavioral difficulty questionnaires, and (computerized) tests assessing concentration, attention, and memory skills. Additionally, patients underwent neurologic, pneumologic, gastrointestinal, and cardiologic assessments.

**Results:**

Overall, the majority of patients reported experiencing elevated levels of fatigue (82.4%), loss of motivation (72.9%), concentration and attention deficits (71.8%), a worsened mood (53%), and a higher level of anxiety (31.8%). The most common diagnosis was the post-COVID adjustment disorder (ICD-10 F43.2, U09.9!; 38.8%) followed by the post-COVID attention deficit disorder (ICD-10 F98.80, U09.9!; 23.5%). Neuropsychiatric evaluation primarily identified deficits in sustained attention. There was a significant association between somatic and psychiatric post-COVID diagnoses. Patients with allergies exhibited a higher risk of developing a post-COVID adjustment disorder. For the post-COVID attention deficit disorder, age, sex, obesity, pre-existing psychiatric diagnosis, and the virus variant were relevant factors.

**Conclusions:**

Our findings indicate a diverse array of neuropsychiatric symptoms associated with the post-COVID syndrome, emphasizing the interconnectedness between somatic and neuropsychiatric diagnoses. To optimize treatment, comprehensive strategies involving both somatic and psychiatric professionals are crucial for addressing the syndrome’s complexity and managing symptoms effectively.

**Study registration:**

The study *Post-COVID Kids Bavaria* was registered with the German Clinical Trials Register (DRKS), funded by the Bavarian State Ministry of Health, Care and Prevention and approved by the Ethics Committee of the University of Regensburg on the 29th of November, 2021 (Reference: 21-2691-101).

## Introduction

Whereas children and adolescents often show mild symptoms during the acute phase of the COVID-19 infection, some develop strong and long-lasting post-COVID symptoms such as headaches and abdominal pain, deficits in concentration and attention, and fatigue [1]. Post-infection symptoms lasting for 4 weeks are termed long COVID and symptoms lasting for more than 12 weeks are termed post-COVID syndrome (PCS) according to the diagnostic criteria of the ICD-10-Code U09.9! post-COVID-19 condition, unspecified [2]. This can include (1) symptoms that do not abate after the acute phase of the infection, (2) newly developed health conditions following the COVID-19 infection, or (3) a deterioration of a preexisting health condition [2]. A diagnosis that is associated with the COVID-19 infection is marked by the addition U09.9! in the diagnostic system ICD-10 published by the WHO [2, 3]. Previous studies mainly focused on the PCS in adults and the somatic sequelae of the infection [4, 5]. Less is known about the symptoms of PCS in children and adolescents, especially the emotional and cognitive components of the syndrome, which is an important research gap to be investigated.

Based on an analysis of 14 studies investigating PCS in children, a wide range of estimated prevalences was evident, spanning from 4 to 66%, and attributable to variations in study methodologies and case definitions [1]. Five of the studies had a control group consisting of children without a COVID-19 infection in the past. Among these, three could show a higher prevalence of lasting symptoms in the patient group compared with the control group, whereas two could not find a difference. It was suggested that the missing statistical difference between children with and without a previous COVID-19 infection could be due to general pandemic effects and not related to the infection itself [6]. There is evidence that pandemic effects also exist for other neuropsychiatric diseases like the pediatric acute-onset neuropsychiatric syndrome (PANS), for example, where sleep disturbances and emotional liability could best explain the increase of symptoms during lockdown [7]. Other statistically significant factors included new symptoms, like depressed mood and eating problems, as well as pandemic-related stress. Conversely, caregivers’ perception of the efficacy of coping strategies was associated with a reduced risk of symptom exacerbation [7].

Potential risk factors for PCS in children include female sex, older age, allergies, and underlying mental and physical health issues before the infection [1, 8]. In addition, the severity and the number of symptoms during the acute phase of the infection are also associated with a higher risk of developing lasting symptoms [1, 8, 9]. Fatigue, headaches, sleep disturbances, cognitive deficits (attention deficits, irritability), respiratory issues (dyspnea), loss of taste and smell, mood disruptions, chest pain, and muscle weakness emerge as recurrent symptoms reported across multiple studies [1, 10, 11, 12]. While some symptoms are consistently observed, certain studies shed light on unique manifestations not extensively mentioned elsewhere. Zimmermann et al. [1] highlighted symptoms such as stomach aches, myalgia, colds, cough, loss of appetite and weight, and rashes. Roge et al. [10] pointed out the occurrence of nocturnal sweating and fever, which were not extensively discussed in other studies. Lopez-Leon et al. [11] emphasized persistent dyspnea, anosmia/ageusia, and fever as distinctive symptoms. Stephenson et al. [12] noted shortness of breath as a notable symptom. Additionally, Borch et al. [13] highlighted dizziness as a unique manifestation in children with PCS. In most cases, the symptoms disappeared after 1–5 months [13]. While most studies report similar PCS symptoms for adults and children/adolescents [14], an analysis of data from the statutory health insurance system in Germany highlighted notable differences. Among children and adolescents, the most common symptoms include malaise, tiredness and exhaustion, cough, as well as chest and cervical pain. In contrast, adults predominantly experience loss of smell and taste, fever, and dyspnea [15]. Additionally, children and adolescents generally have a better prognosis and faster recovery compared to adult populations [16].

### Emotional and neurocognitive long-term effects of COVID-19

The gap between children and adolescent vs. adult literature becomes even more evident regarding the emotional and neurocognitive long-term effects of COVID-19. Although the number of studies with samples comprising children and adolescents is rising, most results are still focused on adults, especially for the underlying risk factors and pathomechanisms. However, there is evidence that both the clinical manifestation and the pathomechanisms for PCS seem to be similar for both populations [14].

A review of adult patients revealed anxiety, depression, and post-traumatic stress disorder as the most common psychological symptoms following a COVID-19 infection, with varying prevalences [17]. Female sex and a higher general pandemic burden were named as possible risk factors for developing psychological sequelae [18]. There is evidence of an association between higher inflammation values and the development of neuropsychiatric post-COVID symptoms [20]. Neuropsychiatric symptoms include attention and memory deficits and deficits in executive functioning [20]. Risk factors for neuropsychiatric symptoms included neurological symptoms, especially headache, and diarrhoea as acute symptoms of the infection [20]. To examine the impact of psychological factors prior to COVID-19 infection on post-COVID symptoms, data was collected through questionnaires assessing depression, anxiety, pandemic-related distress, general stress levels, and perceived loneliness [21]. The findings revealed associations between these factors and a heightened risk of post-COVID symptoms. Notably, elevated scores on two scales were linked to a 50% increased risk [21].

Cognitive aftereffects such as concentration and attention deficits were linked to a strong immune reaction, a cytokine storm, as a possible underlying cause of neurocognitive deficits [19, 22]. C-reactive protein and levels of D-dimers were 6 times higher in adults with severe symptoms of COVID-19, which increases the risk of cerebrovascular complications [23]. Higher levels of D-dimers were correlated with delayed memory and reduced psychomotor activity [24]. Furthermore, there was an association between pulmonary dysfunction and cognitive deficits/deficits in executive functioning [24]. The COVID-19 infection was generally associated with long-term attention/memory deficits and deficits in executive function [20].

A narrative review concerning cognitive and psychiatric post-COVID symptoms in children and adolescents consisting of 102 studies found concentration and memory deficits, sleep disturbances, and anxiety to be the most common symptoms [25]. A meta-analysis showed that there was a higher risk of children developing, inter alia, cognitive deficits, headaches, and dysosmia after a COVID-19 infection than there was in the control group [26]. There was no difference regarding stomach aches, cough, fatigue, myalgia, sleep disturbance, diarrhoea, fever, dizziness, and shortness of breath. The authors reported a pattern in that the higher the quality of the study, the lower was the prevalence of the symptoms. Other findings suggest that there could also be a link between the COVID-19 infection and PANS with post-infectious, autoimmune, and neuro-inflammatory events as the main underlying mechanisms [27]. The authors reported two cases of children who developed symptoms which are typical for PANS, such as the sudden onset of obsessive-compulsive disorder (OCD) or a severely restricted food intake approximately two weeks after the COVID-19 infection [27].

A total of 50 children and adolescents aged 3–12 years were assessed with the Wechsler Intelligence Test for children after a COVID-19 infection [28]. The results were compared with those in a healthy control group (*N* = 20). The children with the previous COVID-19 infection showed significantly worse scores in all tests. The authors concluded that post-COVID symptoms included deficits in verbal–logical thinking, attention focusing, and working memory, which were confirmed in the clinical impression. Conducting a neuropsychological test battery with 25 children and adolescents after a COVID-19 infection and using the results of school children before the pandemic as a control group, the children with the previous COVID-19 infection had significantly worse results in the domains of memory, attention, visual object recognition, visual–spatial processing, kinesthetics, and verbal and nonverbal thinking [29].

In a multidisciplinary post-COVID clinic, 18 pediatric patients were examined and showed results below average in the domain of auditive attention [30]. More than half of the patients with results below average already had mental health problems before the infection, so no causal inference regarding the COVID-19 infection could be made. The most stated symptoms from the patients’ parents were concentration deficits (83.3%), fatigue (77.7%), and sleep disturbances (77.77%). Among 15 parents, 14 reported concerns regarding their children’s mood, and 12 about higher levels of anxiety. Pediatric post-COVID patients as compared with a control group showed significantly more symptoms of an attention deficit disorder [31]. Predictors for limitations in everyday functioning level were reduced goal orientation, depression, anxiety, and attention deficits. In multiple hierarchical regressions controlling for mood and anxiety, anxiety was the only predictor for limitations in the patients’ functional levels, and the authors concluded that therapeutic interventions aiming towards coping techniques for anxiety could reduce the negative effects of the reported cognitive deficits [31].

### Post-COVID syndrome in children compared with sequelae of other infectious diseases

In a study by Roge et al. [10], a comparison was made between children with a COVID-19 infection and children who had another infectious disease. The findings revealed that long-term effects such as fever, fatigue, dysosmia, headaches, cognitive deficits, and nocturnal sweating were significantly associated with a COVID-19 infection, but not with other types of infection. Moreover, 53% of children with a COVID-19 infection experienced two or more long-term symptoms, with the most prevalent being fatigue (25.2%), cognitive deficits such as elevated irritability (24.3%), mood disruption (23.3%), and headache (16.9%). In a retrospective cohort study, data from worldwide patient records from 1,487,712 children, adolescents, and adults with a previous COVID-19 infection were compared with those of patients with other infectious diseases with respiratory interference [32]. The risk of a psychiatric diagnosis was the same for both groups after 1–2 months, but the risk of developing cognitive deficits, psychotic symptoms, and epilepsy was still elevated 2 years after the COVID-19-infection. Children specifically are at a higher risk of developing cognitive deficits and sleep disturbances after a COVID-19 infection, whereas no differences could be found for mood dysregulation and anxiety [32].

### Special features of the immunological profile after a COVID-19 infection

Analyses of the immunological profiles of children and adolescents with and without long-term effects of the COVID-19 infection showed that patients without long-term symptoms showed B-cell homeostasis, which was not the case for post-COVID patients [33]. Additionally, post-COVID symptoms were associated with higher levels of IL6 and IL1β, which influence inflammatory processes and auto-immune reactions and could therefore explain systemic symptoms such as fatigue, post-exertional malaise, headaches, muscle and joint pain, and tachycardia. A recent meta-analysis showed that PCS in adults is characterized by an activation of the immune inflammatory response system and increased immune-associated neurotoxicity with PCS patients showing significantly elevated levels in C-reactive protein and 19 different cytokines compared to controls [34].

The proportions of post-COVID patients who suffered from fatigue (32%) and cognitive deficits (22%) were estimated in a meta-analysis [35]. A following narrative analysis showed these were associated with increased inflammation values. In general, cognitive deficits as long-term effects of COVID-19 infection seem to be connected to hypometabolism in the medial temporal lobe (amygdala, uncus, parahippocampal gyrus) and the pons and cerebellum and a general reduction in grey matter. Those changes are in turn associated with increased inflammation values [36].

### The post-COVID Kids Bavaria study

The Post-COVID Kids Bavaria study [37] was designed to detect early indicators of PCS in children and adolescents. It employs both somatic and psychiatric diagnostic methods to comprehensively characterize the different symptoms of PCS in this population, including emotional and cognitive consequences of the infection. To achieve this, we used infection-specific patient interviews, psychopathological assessments, and a detailed (neuro-) psychological test battery, encompassing computerized assessments of attention and various questionnaires for patients and their guardians. We aim to investigate the transferability of risk factors identified in previous studies to cognitive and emotional post-COVID symptoms in children and adolescents. Our findings will inform the development of suitable multiprofessional diagnostic and treatment approaches that address the (neuro-) psychological long-term effects of the infection, aiming to support affected patients as early and holistically as possible. While epidemiologic studies with a high number of participants regarding the effects of the COVID-19 infection in children and adolescents have been conducted, there remains a gap in systematic observational studies with clinical outpatient populations that employ a multidimensional diagnostic approach combining several medical disciplines. Our study aims to fill this research gap.

## Methods

### Participants and diagnostic procedure

Inclusion criteria for the present observational study were a confirmed COVID-19 infection, ages between 0 and 18 years, and persisting post-COVID symptoms. The COVID-19 infection was assessed via at least one of the following methods: PCR test (91.9%), antigen test (15.1%), and one or more confirmed COVID-19 infections in the family (26.7%). Participants were recruited through a network of paediatricians and general practitioners within the region and contacted by a study assistant to confirm inclusion criteria and schedule appointments. Our analyses are based on patients who were assessed in our post-COVID clinic in Regensburg. Prior to data collection, the study was approved by the ethics committee of the University of Regensburg (Reference: 21-2691-101, approved on 29th of November, 2021) and registered in the German Clinical Trials Register (DRKS00028742). A general checklist for preexisting health conditions was completed by each patient’s parents and paediatrician in advance. Upon obtaining informed written consent for participation, participants also completed a questionnaire regarding the timeline of the coronavirus infection and associated symptoms and provided evidence of the infection. The medical history interview, encompassing categories such as the timeline of infection, acute/long-term symptoms, and deterioration indicators (tiredness, quality of sleep, fatigue, appetite, eating habits, mood, anxiety, irritability, concentration, thought disorders), was conducted as the initial phase of the child and adolescent psychiatric examination. This examination was performed by clinical psychologists well trained in child and adolescent psychiatry. Subsequently, the guardians were handed the questionnaires and asked to complete them in the absence of the researchers. The questionnaires included sociodemographic information, the Strengths and Difficulties Questionnaire (SDQ, with modifications to assess pre- and post-COVID-19 infection evaluations) [38], the COVID-19 Questionnaire General (COV-GEN) (a modified version of The German COVID-19 Questionnaire for Anorexia Nervosa (COV-AN) [39]) to estimate general pandemic effects (encompassing changes in family dynamics, hobbies, social interactions, media consumption, isolation, school, eating habits and sleeping patterns), the WHO-5 questionnaire [40] for general well-being and, in case of signs of chronic fatigue syndrome, a screening for post-exertional malaise (PEM) [41]. Following the Arbeitsgemeinschaft für Methodik und Dokumentation (AMDP) manual [42], the psychopathological examination and (neuro-) psychological assessment commenced. The AMDP system is a tool for documenting psychiatric symptoms in clinical and research projects and offers a clear structure for identification and categorization of psychopathological symptoms [42]. To detect attention and memory deficits, we used the subtests Go/NoGo (impulse control), Incompatibility (selective attention), and Visual Scanning (sustained attention) of the computerized attention assessment system TAP/KiTAP [43]. Additionally, we utilized the subtest digit span from the Wechsler Intelligence Test for Children (WISC-V) [44] to assess working memory. Children aged 10 and above also completed the WHO-5 questionnaire assessing general well-being and the Emotional Symptoms scale of the SDQ (with Pre- and Post-COVID scores). The questionnaires were incorporated into the study in March 2022, resulting in a smaller sample size than the total cohort. After the neuropsychological assessment (lasting approximately 1 h), patients underwent four additional examinations (cardiovascular, pulmonary, neurological, and gastroenterological assessment, lasting approximately 4 h).

Following the clinical assessment, the results were then discussed with a multidisciplinary team of clinical psychologists and physicians, and diagnoses were assigned following the ICD-10 classification system [3] and the AWMF guidelines [2]. When the symptoms were clearly linked to the COVID-19 infection and were therefore classified as PCS, and the addition “U09.9!” was documented. Then, patients were informed on test results and provided with recommendations for further treatment such as psychotherapy, rehabilitation, or further diagnostic procedures.

### Statistical analysis

On the basis of previous research, we investigated the following risk factors for emotional problems: the number and severity of symptoms during the infection, sex, age, asthma, allergies, obesity, previous health conditions, a higher pandemic burden measured by the questionnaire COV-GEN, a higher psychological burden measured by psychosocial circumstances, and the virus variant (Delta vs. Omicron). Emotional outcome measures were diagnoses, the difference in pre/post scores in the SDQ questionnaire, a WHO wellbeing score under 50, which is associated with symptoms of depression [40], number of deteriorations in the patient interview and psychological symptoms in the psychopathological examination, and scores below average in the neuropsychological test battery. Cognitive risk factors included neurological symptoms during the acute phase of the infection including headache, diarrhoea, and higher inflammation values. Cognitive outcome variables were the diagnosis of a post-COVID attention deficit disorder, scores below average in the attention and memory tests, and attention deficits in the psychopathological examination as well as the patient history interview. In a first step, we conducted correlational analyses (in case of metric variables) or a chi-square test (for nominal variables) between the risk factors and outcome variables. In a second step, we conducted a linear, ordinal, or logistic regression analysis controlling for sex and age and including risk factors with significant correlations as predictors. To test our hypothesis regarding higher scores after the infection than before the infection in the SDQ questionnaire, we used Wilcoxon tests. The level of significance was set to *p* ≤ 0.05. The *p*-values were adjusted for multiple comparisons using the False-Discovery-Rate method [45]. To assess the relationship between child and adolescent psychiatric and somatic diagnoses, the chi-square test was used.

## Results

We examined the results of 85 children and adolescents with a mean age of 12.48 years (*SD* = 3.12) and an age span from 2 to 17 years of age; 61.2% were female. Two patients were excluded from the analysis in advance, one because their symptoms were not clearly linked to a COVID-19 infection and another because the questionnaires were incomplete. The mean time between the infection and the examination was 5.7 months (*SD* = 4.98). The data collection took place from December 2021 to June 2023. Regarding education, 4.7% attended preschool, 20.0% were in primary school, and 76.3% attended secondary school (36.5% in the highest level of education in Germany).

**Fig. 1:**
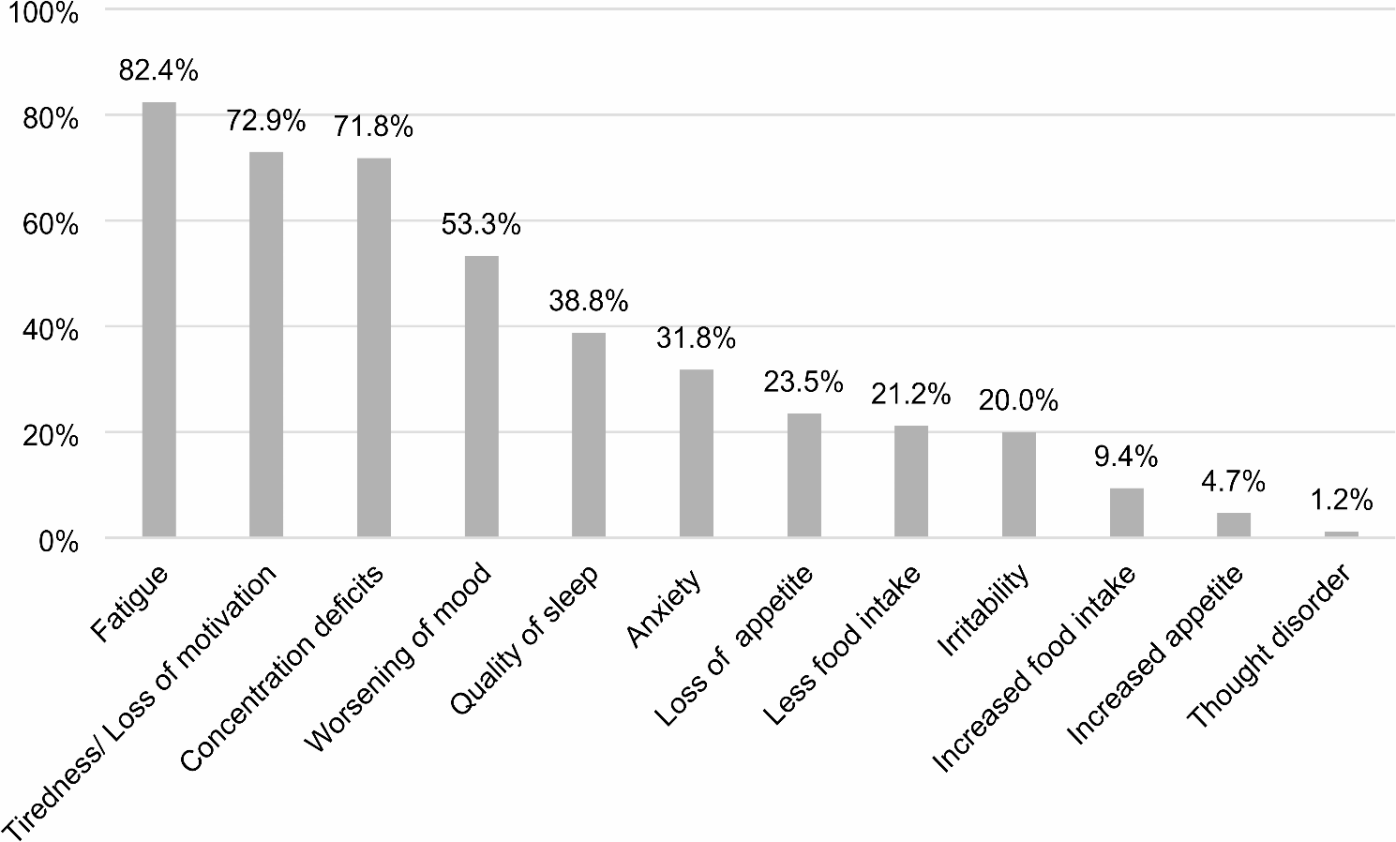
Percentages of patients reporting a deterioration in the different categories of the patient history interview following COVID-19 infection (*N* = 85)

**Fig. 2:**
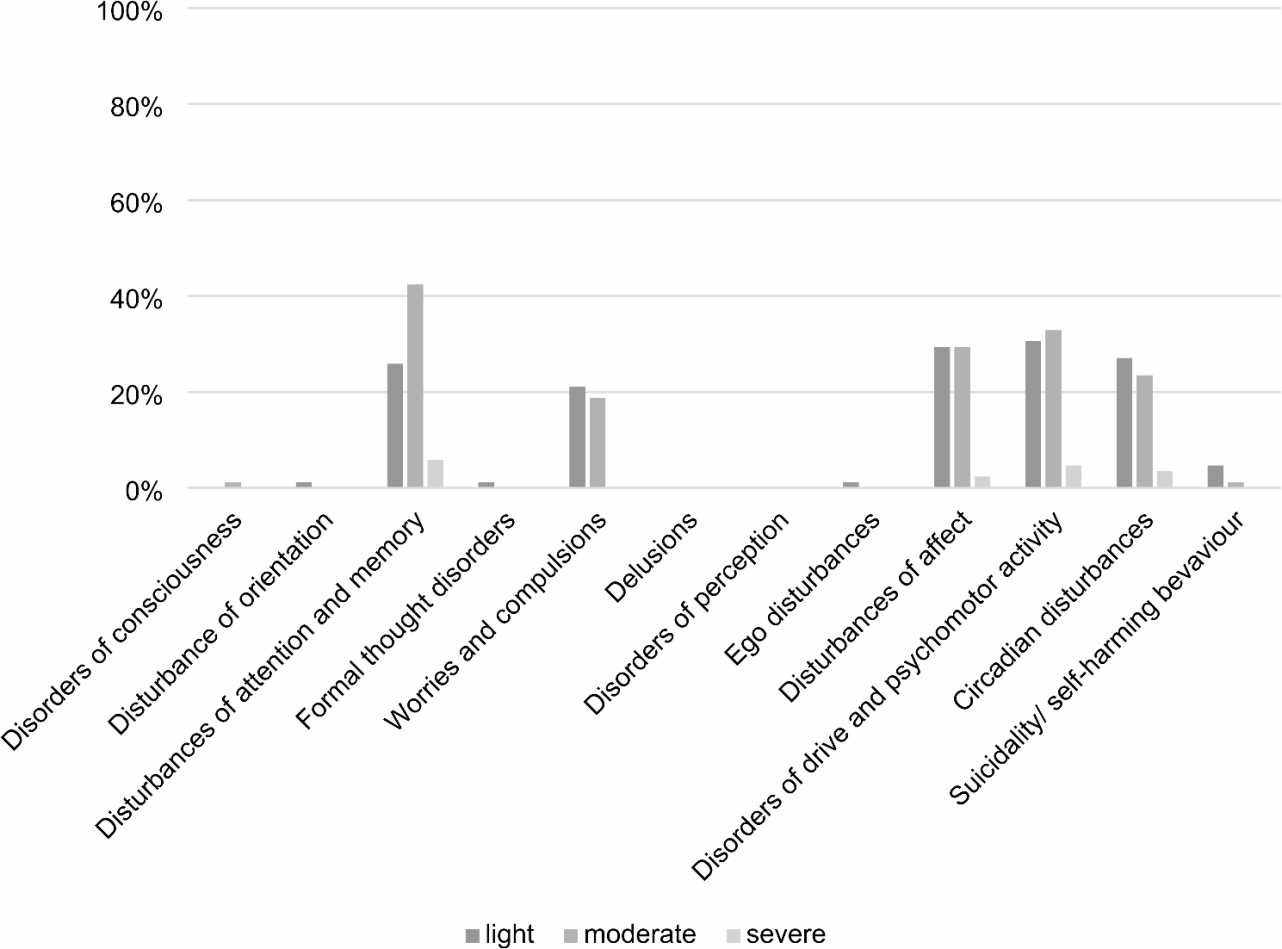
Percentage of patients (*N* = 85) marked as lightly, moderately, or severely affected by psychopathological disturbances based on our psychopathological examination according to the AMDP^1^ System ^1^Arbeitsgemeinschaft für Methodik und Dokumentation in der Psychiatrie. The differentiation between light, moderate and severe abnormalities was implemented according to the diagnostic guidelines in the AMDP Manual

A descriptive analysis (see Fig. 1) of the patient history interview, the psychopathological examination, the questionnaires, and the neuropsychological assessment revealed a higher level of fatigue (82.4%), lack of motivation (72.9%), concentration deficits (71.8%), and worsening of mood (53.3%). In the psychopathological examination, the four categories above normal thresholds (mild, moderate, severe) for most patients were disturbances in attention and memory, drive and psychomotor activity, affect, circadian disturbances, and worries and compulsions; the detailed results are illustrated in Fig. 2. Overall, the psychopathological examination was marked as abnormal for 83.5% of our patients.

More than half of the examined children and adolescents (54.21%) were strongly advised to undergo further child and adolescent psychiatric diagnostic assessment, 30.20% were given optional advice for further diagnostics, 12.49% were given other advice like psychotherapy, further somatic assessment, regular weight control, and specialized diagnostics for chronic fatigue syndrome or occupational therapy; for 3.1% there were no further recommendations.

**Fig. 3:**
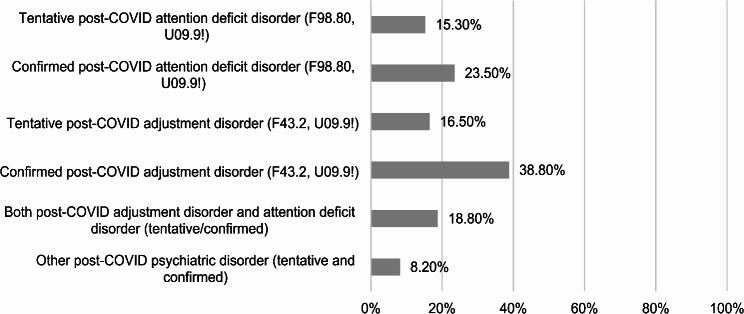
Percentage of patients (*N* = 85) with the respective post-COVID diagnoses. As patients could meet the criteria of more than one diagnosis, the percentages do not add up to 100%

The analysis of the assigned child and adolescent psychiatric diagnoses showed that 14.1% had no diagnosis at all, 8.3% had only a child and adolescent psychiatric diagnosis that was not associated with the COVID-19 infection, and 56.47% had a confirmed and 21.13% had a suspected post-COVID psychiatric diagnosis. The most common diagnoses were post-COVID adjustment disorder (F43.2 with U09.9!) and post-COVID attention deficit disorder (F98.80 with U09.9!). Other diagnosed psychiatric disorders included depression and social phobia, which were very rare. Altogether, 28.2% of the patients had a preexisting child and adolescent psychiatric diagnosis. For a detailed illustration of the specific diagnoses, see Fig. 3. A comorbid child and adolescent psychiatric diagnosis such as ADHD, depression, or different kinds of phobia before the COVID-19 infection was stated for 20% of patients with a psychiatric post-COVID diagnosis.

The neuropsychological assessment included the WISC-V digit span (*N* = 84, one patient was too young to be assessed), in which 11.8% of the patients showed a result below average, 82.4% had average results, and 4.7% had results above average (*M* = 9.42, *SD* = 2.47). The results of the computerized attention assessment (KiTAP/TAP) showed mainly average results except for reaction times and omissions in the subtest Visual Scanning, see Table 1. The number of assessed patients varies because of missing norms for younger patients and technical difficulties.

**Table 1:** Results of the neuropsychological assessment with the computerized test battery for the assessment of attentional functioning, TAP^1^

Subtest	Variable	Below average	Average	Above average
Go/NoGo (*N* = 82)	Reaction Time	22.22%	66.67%	11.11%
	Mistakes	15.85%	70.73%	13.41%
	Omissions	14.63%	85.73%	0.00%
Visual Scanning (*N* = 73)	*Critical Trials*: Reaction Time	41.43%	48.57%	10.00%
	*Critical Trials: *Omissions	50.00%	45.95%	4.05%
	*Noncritical Trials*: Reaction Time	27.40%	64.38%	8.22%
	*Noncritical Trials:* Mistakes	16.44%	83.56%	0.00%
Incompatibility (*N* = 81)	Reaction Time	29.63%	69.14%	1.23%
	Mistakes	12.35%	38.27%	49.38%

^1^ Testbatterie zur Aufmerksamkeitsprüfung (TAP)

In the WHO well-being index, the mean score of the patients (*N* = 55) was 11.44 (*SD* = 5.27), and the main percentage score was 45.75 (*SD* = 21.09). Scores under 12 and percentage scores under 50 are associated with depression [34]. The mean in the parents’ evaluation (*N* = 65) was 11.78 (*SD* = 5.99) with a mean percentage score of 46.11 (*SD* = 22.13). The mean estimations of the COV-GEN (*N* = 64), varying from − 3 (highest degree of deterioration) to 3, were − 0.23 (*SD* = 0.84) for friends, − 0.37 (*SD* = 0.82) for hobbies, − 0.39 (*SD* = 1.26) for isolation, − 0.34 (*SD* = 1.18) for family, − 1.42 (*SD* = 1.00) for media consumption, − 0.15 (*SD* = 0.72) for school, − 0.39 (*SD* = 1.23) for quality of sleep, and − 0.67 (*SD* = 0.95) for eating habits. The PEM-Screening (*N* = 56) was positive for 69.6%: 23.2% stated that exhaustion after any kind of activity lasted for more than 14 h, 57.1% were more exhausted than usual after a small amount of mental activity, 67.9% were more exhausted than usual after a small amount of physical activity, and 53.6% were more exhausted than usual after both kinds of activity. There was a significant increase across all problem scales of the SDQ and a decrease in scores on the Prosocial Behavior scale of after the infection as compared with the pre-COVID scores (see Table 2). Patients and their guardians were asked to fill out the questionnaire by providing retrospective estimations for the period prior to the COVID-19 infection and assessments for the period following the infection.

**Table 2:** Analyses of pre- and post-COVID scores in the Strengths and Difficulties Questionnaire^1^

Scale	Score pre-COVID	Score post-COVID	Z-value	*p*-value	Cohen’s d	
*Self-assessment* (*N* = 55) Emotional symptoms	1.69	3.89	−5.727	< 0.001	0.71	
*Guardians’ assessment* (*N* = 65): Emotional symptoms	1.67	4.16	−6.314	< 0.001	0.79	
*Guardians’ assessment(N = 65): *Conduct symptoms	1.42	1.91	−2.808	0.005	0.35	
*Guardians’ assessment(N = 65): *Hyperactivity/inattention	2.22	3.75	−5.410	< 0.001	0.68	
*Guardians’ assessment(N = 65): *Peer relationship problems	1.36	2.02	−3.977	< 0.001	0.50	
*Guardians’ assessment(N = 65): *Prosocial behavior	8.72	8.31	−3.209	0.001	0.40	
Total score (problem scales, without prosocial behavior)	6.66	11.91	−6.462	< 0.001	0.81	

^1^ Strengths and Difficulties Questionnaire (SDQ)

For the outcome variable secured post-COVID diagnosis, the result of the logistic regression was significant for the factors sex, age, preexisting health condition, and the score on the family scale of the COV-GEN questionnaire (*χ2* [4] *=* 12.012, *p* = 0.017, R^2^ = 0.230), correctly predicting 70.3% of the cases. However, none of the factors alone was a significant predictor (all *p* > 0.085). For the categories of the psychopathological examination that were marked as abnormal, we found older age and a preexisting health condition to be significant predictors in a linear regression (*F*[8,52] = 3.024, *p* = 0.007). The detailed results of all linear regressions are illustrated in Table 3. Also, taking into account the severity of the psychopathological categorization, older age and deterioration on the COV-GEN scale Isolation were significant predictors in linear regression (*F*[5,55] = 3.585, *p* = 0.007; details in Table 3). Also in a linear regression, we found deterioration on the COV-GEN scale Quality of Sleep to be a significant predictor of a higher pre-/post-COVID difference on the SDQ scale Peer Relationship Problems (*F*[3,60] = 2.864, *p* = 0.044; Table 3). A significant ordinal regression analysis (R^2^ = 0.204, *p* = 0.022) with the outcome variable Pre/Post Difference of the classification regarding the scale Hyperactivity/Inattention showed that deterioration on the COV-GEN scale Isolation was associated with a higher probability of switching from a higher to a lower category after the infection, with an odds ratio of 0.410 (95% CI 0.202 to 0.834, Wald *χ*^2^ [1] = 6.065, *p* = 0.014). There were no significant results in the analysis of risk factors for neurocognitive deficits. There was a significant association between somatic diagnosis and child and adolescent psychiatric diagnosis (*X*^2^ [1, *N* = 85] = 4.276, *p* = 0.039), especially for post-COVID diagnosis from the neurological department (e.g., chronic headache, anosmia) (*X*^2^ [1, *N* = 85] = 6.807, *p* = 0.009). In a logistic regression analyzing possible risk factors for developing a post-COVID adjustment disorder, we found having a preexisting allergy (*p* = 0.047, B = 1.265) to be a significant predictor (*χ2* [3] *=* 9.931, *p* = 0.017, R^2^ = 0.150), correctly predicting 68.2% of the cases. The other factors included sex (*p* = 0.176) and age (*p* = 0.074). For the post-COVID attention deficit disorders there was a significant logistic regression for the combination of age, sex, virus variant, preexisting mental health condition, and obesity (*χ2* [3] *=* 22.919, *p* < 0.001, R^2^ = 0.374), correctly predicting 76.3% of the cases. Again, no factors were significant predictors on their own (all *p* > 0.060).

**Table 3:** Results of linear regression analyses

Dependent variable	Predictor	B	SE	Β	T	*P*
Psychopathological examination (number of categories marked as abnormal)	Sex	− 0.030	0.392	− 0.009	− 0.076	0.940
	Age	0.137	0.063	0.266	2.185	**0.033**
	Virus variant	1.263	0.659	0.254	1.917	0.061
	Preexisting health condition	0.818	0.378	0.255	2.166	**0.035**
	Treatment setting during infection	0.014	0.247	0.008	0.058	0.954
	Obesity	− 0.129	0.289	− 0.057	− 0.446	0.657
	COV-GEN^1^: isolation	− 0.322	0.167	− 0.257	−1.930	0.059
	COV-GEN^1^: quality of sleep	− 0.115	0.160	− 0.090	− 0.718	0.476
Psychopathological examination (severity of categories marked as abnormal)	Sex	0.240	0.722	0.040	0.332	0.741
	Age	0.243	0.115	0.252	2.115	**0.039**
	Preexisting health condition	1.176	0.706	0.196	1.666	0.101
	Virus variant	1.789	1.149	0.192	1.558	0.125
	COV-GEN^1^: isolation	− 0.641	0.292	− 0.273	−2.198	**0.032**
SDQ^2^ Pre-/Post-Covid difference on the scale Peer relationship problems	Sex	− 0.101	0.299	− 0.042	− 0.336	0.738
	Age	0.033	0.047	0.086	0.709	0.481
	COV-GEN^1^: quality of sleep	− 0.334	0.121	− 0.343	−2.768	**0.007**

^1^COVID-19 General Questionnaire ^2^ Strengths and Difficulties Questionnaire (SDQ)

## Discussion

Analyzing the neuropsychiatric symptoms of PCS in children and adolescents, we found that fatigue, loss of motivation, attention and memory deficits, and affective disorders were the most common, in line with previous findings [11]. The psychopathological examination resulted in 83.5% of patients being rated as abnormal, therefore clearly surmounting the point prevalence of mental illness among children and adolescents (15%) [46] or the COPSY study reporting 30% of children and adolescents in Germany showing psychological abnormalities during the pandemic [47]. More than 50% of our patients had a psychiatric post-COVID diagnosis, with the post-COVID adjustment disorder and post-COVID attention disorder being the most prevalent. Cognitive deficits were mostly evident in the domain of sustained attention. Patients reacted more slowly and had more omissions than expected compared with the Gaussian distribution in the subset Visual Scanning, which lasted longer than 10 min. In contrast, previous studies [28, 29] showed a broader range of neurocognitive deficits that could not be confirmed in our neuropsychological tests. This could be due to missing pre-COVID data, as average results could also represent a deterioration relative to a pre-COVID baseline. Additionally, the lack of a control group limits our ability to isolate COVID-specific effects and interpret the data conclusively. Generally, it is important to note that the rate of pre-diagnosed psychiatric diagnoses in our sample is higher than the expected point prevalence of psychiatric diagnoses of children and adolescents which is estimated to be 15% [46]. This discrepancy could be explained by several factors. First, there may be a selection bias, as children and adolescents with pre-existing psychiatric diagnoses—who likely have prior experience seeking help and receiving treatment—might be more inclined to seek medical care in general, leading to their overrepresentation in our sample. Second, previous psychiatric diagnoses have been identified as a risk factor for developing post-COVID syndrome [1, 8, 48], which may also contribute to the higher rate observed. Finally, the increased prevalence of psychiatric diagnoses during the pandemic may also play a role, reflecting the broader mental health impacts of the pandemic itself [49].

Patients showed significantly higher scores on all problem scales and significantly lower scores on the Prosocial Behavior of the SDQ after the infection. Therefore, a COVID-19 infection seems to have an impact on a wide range of psychological dimensions, as observed in previous studies [25, 30]. This is also evident in the WHO-5 well-being index, with a mean score below 12 and more than 50% of the patients in a scoring section that is associated with symptoms of depression. It is still unclear whether the psychological sequelae are a primary or secondary effect of the infection, although previous studies suggest that both could be the case [50, 51]. Patients scheduling appointments at a post-COVID clinic may exhibit cognitive biases when completing questionnaires, potentially attributing symptoms to a COVID-19 infection, thus creating a self-fulfilling prophecy. Nonetheless, disparities observed in pre-/post-COVID SDQ scores align with insights acquired from comprehensive patient interviews, pinpointing deteriorations likely emerging subsequent to a COVID-19 infection.

There is evidence that pandemic effects play a role in the development of neuropsychiatric long-term effects after a COVID-19 infection, but only in very specific areas and for specific outcome variables. Our data suggests that changes in relationship and activity patterns in families, such as more/fewer conflicts and more/less time spent together could, in combination with age, sex, and preexisting health conditions, lead to a higher risk of developing a child and adolescent psychiatric post-COVID diagnosis. However, the regression model did not show significant individual predictors despite overall model significance, so directional interpretations are not possible and further studies with bigger sample sizes might be necessary to find individually significant predictors stating the direction of the interrelation.

Furthermore, being more isolated during the pandemic was a significant predictor of stronger and/or more anomalies in the psychological examination as well as a lower category on the SDQ scale Hyperactivity/Inattention post compared with pre-COVID-19 infection. Deterioration in the quality of sleep was significantly associated with a higher pre-/post-COVID difference on the SDQ subscale Peer Relationship Problems. It is important to note that pandemic restrictions were abolished during the period of data collection but could still have aftereffects on children and adolescents [47]. The COPSY study [47] showed an increase in the number of children and adolescents with mental problems from 22 to 30% during the pandemic. These percentages are higher in our study regarding questionnaire results, the proportion of post-COVID psychiatric diagnoses and abnormalities in the psychopathological examination, which counteracts the possibility that PCS is merely a reflection of pandemic effects. This is further supported by higher percentages of children and adolescents scoring in the category abnormal and borderline in the SDQ in our study compared to the COPSY study examining pandemic effects on children and adolescents, especially on the scale emotional problems (20–25% vs. 56.3%) [47]. The significant pre-/post-COVID differences in the SDQ questionnaire also suggest that the symptoms are linked to the COVID-19 infection and not due to pandemic effects. This is in line with the results from the patient interview and our clinical observations, where some of the children and adolescents were so impaired after the COVID-19 infection that they could not go to school or fulfill their daily tasks anymore which could not be explained by other factors. In general, our findings do not confirm that post-infectious symptoms are merely general pandemic effects as previously suggested [6], but a pandemic burden in some areas can partly be an impact factor for contribute to the development of cognitive and emotional post-COVID symptoms, in line with the findings of Wang et al. [21]. We could not find significant predictors for existing neurocognitive deficits, possibly due to the low number of patients with diarrhoea during the acute phase of the infection and the high number of missing inflammation values. Symptoms during the acute phase of the infection could also be incomplete because of the long period between the infection and the neuropsychological assessment.

There was a statistically significant connection between somatic and psychiatric post-COVID diagnoses, suggesting that these symptom groups are interconnected and that multidisciplinary teams are necessary to diagnose and treat pediatric patients with PCS. Patients with allergies as preexisting health conditions were at a higher risk of developing a post-COVID adjustment disorder, in line with previous studies concerning general risk factors for post-COVID symptoms [8, 9]. However, we did not differentiate between different forms and severity of allergies, which should be included in further research. For the post-COVID attention deficit disorder, the interaction of possible risk factors was more complex: none of the predictors of age, sex, obesity, preexisting mental health conditions, and virus variant were statistically significant on their own, limiting the possibilities for interpretation. This may be attributed to the limited sample size. Follow-up studies with larger participant groups, offering greater statistical power, could yield more robust and statistically significant individual predictors.

Generally, as we examined only patients with existing long-term effects of a COVID-19 infection, the stated risk factors are only valid for this group of patients and they cannot yet be transferred to the general public. As we examined patients with existing long-term effects who were mainly recruited from paediatricians, the results may be subject to allocation bias. These limitations reduce the generalizability of our findings to the broader population, particularly to milder cases of post-COVID syndrome (PCS) in individuals who do not seek medical assistance. As a result, the identified risk factors are primarily applicable to children and adolescents with PCS severe enough to require medical intervention. Further research involving more diverse and representative samples is necessary to enhance the generalizability of these results.

Our study is subject to several limitations, notably the absence of a control group and lack of pre-COVID data for the attention and memory tests and the WHO-5 well-being index. This limits the study’s ability to isolate COVID-19 specific effects and provide causal interpretations. To address these limitations, we introduced a pre-COVID score in the SDQ to evaluate differences in emotional symptoms, comparing the times before and after the COVID-19 infection. However, it is important to acknowledge that response biases may still influence these findings. To counteract this possibility, we conducted very specific anamnesis interviews focusing on changes distinctly beginning after the COVID-19 infection. In order to better distinguish between COVID-19 specific effects and other factors contributing to developing symptoms which are typical for PCS, we suggest implementing follow-up studies with matched controls and/or pre-COVID data concerning neuropsychological assessment and psychological questionnaires.

Despite these limitations, our study fills an existing research gap where data from systematic observational studies with a broader variety of interdisciplinary diagnostic instruments has been missing. Our study presents results not only from questionnaires but also from a thorough psychopathological and neuropsychological examination combined with a broad range of somatic assessments. Together, the results of our study reveal that some children and adolescents, spanning from very young ages to early adulthood, are experiencing significant post-COVID-19 neurocognitive and emotional long-term effects that severely impact their daily functioning. This highlights a substantial proportion of pediatric post-COVID patients who require early intervention through child and adolescent psychiatric diagnostics and treatment. By focusing on identified risk factors such as sleep quality, changes in family dynamics and activities, and isolation, it becomes evident that preventive measures, including raising awareness about these influences and implementing support programs, could help to reduce psychological long-term effects in children and adolescents across the board. It is crucial to not only classify and treat PCS in children and adolescents as a somatic phenomenon but also emphasize the psychological component to offer holistic treatment to young patients, including support for emotional and cognitive sequelae.

## Conclusions

Our results show that there is a wide range of child and adolescent psychiatric symptoms connected to PCS as well as a significant association between somatic and psychiatric post-COVID diagnoses. Therefore, multidisciplinary diagnostic and treatment plans including child and adolescent psychiatric specialists are necessary. Additionally, changes in family dynamics, isolation, and quality of sleep can have an impact on long-term effects, so providing (therapeutic) help in these areas could help prevent or improve symptoms. We found that allergies seem to play a role in the development of a post-COVID adjustment disorder, as do the combination of factors age, sex, virus variant, preexisting health conditions, and obesity in the development of post-COVID attention deficit disorder. However, further research is needed to determine specific risk factors for neuropsychiatric sequelae of the infection as we could analyze only patients who had already developed long-term effects and cannot transfer the found risk factors to the general public.

## Data Availability

The datasets used and/or analyzed during the current study are available from the corresponding author upon reasonable request.

## Abbreviations

ADHD: Attention Deficit Hyperactivity Disorder
COVID-19: Coronavirus disease
OCD: Obsessive-compulsive disorder
PANS: Pediatric acute-onset neuropsychiatric syndrome
PCS: Post-COVID syndrome
PEM: Post-Exertional Malaise
